# Disease-specific T cell receptors maintain pathogenic T helper cell responses in postinfectious Lyme arthritis

**DOI:** 10.1172/JCI179391

**Published:** 2024-07-04

**Authors:** Johannes Dirks, Jonas Fischer, Julia Klaussner, Christine Hofmann, Annette Holl-Wieden, Viktoria Buck, Christian Klemann, Hermann J. Girschick, Ignazio Caruana, Florian Erhard, Henner Morbach

**Affiliations:** 1 Pediatric Inflammation Medicine, Department of Pediatrics, University Hospital Würzburg, Würzburg, Germany.; 2 German Centre for Infection Research (DZIF), Partner Site Hamburg-Lübeck-Borstel-Riems, Lübeck, Germany.; 3 Institute of Pathology, University of Würzburg, Würzburg, Germany.; 4 Department of Pediatric Immunology, Rheumatology, and Infectiology, Hospital for Children and Adolescents, Leipzig University, Leipzig, Germany.; 5 Children’s Hospital, Vivantes Klinikum im Friedrichshain, Berlin, Germany.; 6 Pediatric Hematology, Oncology and Stem Cell Transplantation, University Hospital Würzburg, Würzburg, Germany.; 7 Computational Systems Virology and Bioinformatics, Institute for Virology and Immunobiology, University of Würzburg, Würzburg, Germany.; 8 Faculty for Informatics and Data Science, University of Regensburg, Regensburg, Germany.

**Keywords:** Autoimmunity, Infectious disease, Arthritis, Autoimmune diseases, T cell receptor

## Abstract

**BACKGROUND:**

Antibiotic-Refractory Lyme Arthritis (ARLA) involves a complex interplay of T cell responses targeting *Borrelia burgdorferi* antigens progressing toward autoantigens by epitope spreading. However, the precise molecular mechanisms driving the pathogenic T cell response in ARLA remain unclear. Our aim was to elucidate the molecular program of disease-specific Th cells.

**METHODS:**

Using flow cytometry, high-throughput T cell receptor (TCR) sequencing, and scRNA-Seq of CD4^+^ Th cells isolated from the joints of patients with ARLA living in Europe, we aimed to infer antigen specificity through unbiased analysis of TCR repertoire patterns, identifying surrogate markers for disease-specific TCRs, and connecting TCR specificity to transcriptional patterns.

**RESULTS:**

PD-1^hi^HLA-DR^+^CD4^+^ effector T cells were clonally expanded within the inflamed joints and persisted throughout disease course. Among these cells, we identified a distinct TCR-β motif restricted to HLA-DRB1*11 or *13 alleles. These alleles, being underrepresented in patients with ARLA living in North America, were unexpectedly prevalent in our European cohort. The identified TCR-β motif served as surrogate marker for a convergent TCR response specific to ARLA, distinguishing it from other rheumatic diseases. In the scRNA-Seq data set, the TCR-β motif particularly mapped to peripheral T helper (T_PH_) cells displaying signs of sustained proliferation, continuous TCR signaling, and expressing CXCL13 and IFN-γ.

**CONCLUSION:**

By inferring disease-specific TCRs from synovial T cells we identified a convergent TCR response in the joints of patients with ARLA that continuously fueled the expansion of T_PH_ cells expressing a pathogenic cytokine effector program. The identified TCRs will aid in uncovering the major antigen targets of the maladaptive immune response.

**FUNDING:**

Supported by the German Research Foundation (DFG) MO 2160/4-1; the Federal Ministry of Education and Research (BMBF; Advanced Clinician Scientist-Program INTERACT; 01EO2108) embedded in the Interdisciplinary Center for Clinical Research (IZKF) of the University Hospital Würzburg; the German Center for Infection Research (DZIF; Clinical Leave Program; TI07.001_007) and the Interdisciplinary Center for Clinical Research (IZKF) Würzburg (Clinician Scientist Program, Z-2/CSP-30).

## Abstract 2

Disease-specific T cell receptors from synovial T cells help characterize the pathogenic T cell responses in patients with postinfectious Lyme arthritis.

## Graphical abstract

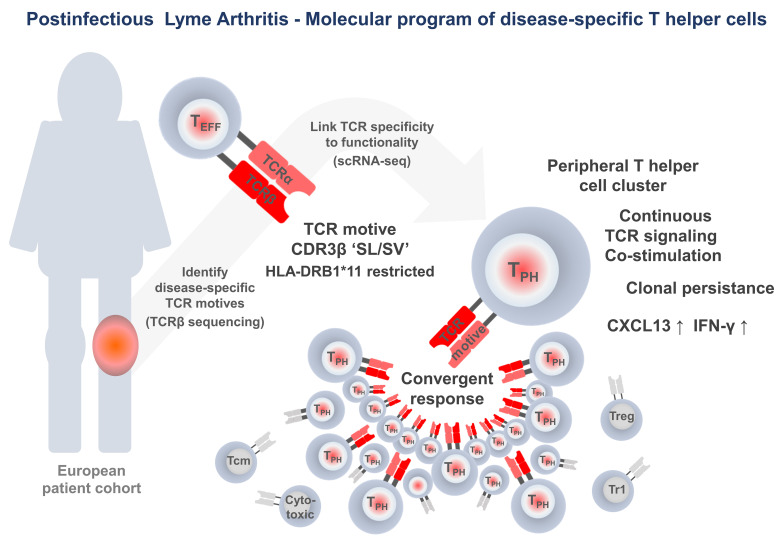

## Introduction

Lyme disease, caused by the tick-transmitted spirochete *Borrelia (B.) burgdorferi* *sensu lato*, is the most prevalent vector-borne illness in North America and Europe, posing a significant public health concern (1, 2). In North America, *B*. *burgdorferi* *sensu stricto* (s.s.) is the main species, while in Europe, additional species such as *B*. *afzelii* and *B*. *garinii* contribute to disease burden (3). Among the late manifestations, Lyme Arthritis (LA) is the most commonly reported (4). While the majority of cases resolve after antibiotic treatment, a small subgroup of individuals experiences persistent joint inflammation despite adequate antibiotic treatment, this condition is referred to as postinfectious or antibiotic-refractory LA (ARLA) (5–8).

ARLA is characterized by an exaggerated proinflammatory immune response that persists after the initial infection and results in synovitis and synovial hyperplasia. Infections caused by more virulent *Borrelia burgdorferi* strains, especially those belonging to the RST1 (OspC type A) genotype, are correlated with the development of ARLA (9). These strains exhibit genetic traits conducive to heightened expression of outer surface lipoproteins, which are suggested to be implicated in inducing exaggerated immune reactions in susceptible individuals (10). In line with this, the *Borrelia burgdorferi* lipoprotein outer surface protein A (OspA) has been identified as a well-established antigen triggering this dysregulated immune response (11–14). The risk of developing ARLA has been linked to specific HLA-DRB1 alleles that efficiently present OspA-derived peptides to CD4^+^ T cells (13, 15). In a North American study, HLA-DRB1 alleles *01:01, *04:01, and *15:01 were associated with a higher risk, while *08:01, *11:01, *11:04, and *13:02 were associated with a potential protective capacity (15). In individuals at risk, OspA appears to trigger an excessive immune response creating a highly inflammatory milieu that subsequently promotes epitope spreading of the immune response toward autoantigens (7). This autoimmune response seems to be mediated by T-bet and/or ROR-γt–expressing Th cells and targeted extracellular matrix proteins as well as vascular autoantigens (16–20).

While this may point toward Th1 and/or Th17 cells as drivers of arthritis, the detailed function and molecular program of pathogenic Th cells in patients with ARLA remain unclear. Moreover, the concept of an OspA-mediated dysregulated immune response might not directly extend to regions outside of North America where the composition of *Borrelia* species causing LA differs. This discrepancy is evident since immune responses against OspA are noticeable in many patients in North America, especially those experiencing ARLA, but they are seldom observed in patients with LA in Europe (21, 22).

Hence, our objective was to ascertain the molecular program of disease-specific Th cells in patients with ARLA, link T cell receptor (TCR) specificity to cellular function, and track clonal evolution of these cells at the site of inflammation throughout disease progression. Acknowledging the uncertainty of potential target antigens among patients outside of North America, we implemented an unbiased approach focusing on the host’s immune response. The objective was to infer putative TCR specificity through the clustering of TCRs displaying biological similarities. For this, we employed high-throughput TCR sequencing of activated CD4^+^ Th cells from the joints of patients in conjunction with scRNA-Seq.

Using this approach, we identified disease-specific TCRs in inflamed joints of patients with ARLA living in Central Europe, mainly restricted to HLA-DRB1*11 or HLA-DRB1*13 alleles, which were unexpectedly prevalent in our cohort despite their underrepresentation in the North American cohort. These ARLA-specific T cells exhibited sustained proliferation, likely due to locally induced TCR signaling, and expressed a pathogenic effector program resembling peripheral Th (T_PH_) cells, marked by CXCL13 and IFN-γ expression.

## Results

### PD-1^hi^HLA-DR^+^CD4^+^ effector cells are expanded in the joints of patients with ARLA throughout disease course.

We studied a cohort of 13 pediatric patients with ARLA (mean ± SD age of onset 13.6 ± 2.2 years; 38.5% female and 61.5% male) residing throughout Germany, from whom synovial fluid (SF) was preserved following therapeutic joint injections after they had completed antibiotic therapy. Antibiotic treatment was initiated within 2 months after onset of arthritis in 7 of these 13 patients (median 2.0 months, range 7 days to 10 months). Despite prior antibiotic treatment, all patients presented with chronic arthritis and received intraarticular steroid injection. All patients were followed for at least 12 months, with a median followup of 21 months (range 12 to 60 months). All but a single patient experienced a recurrence of arthritis after intraarticular steroid injection and required treatment with DMARDs, along with additional intraarticular steroid injections (Supplemental Figure 1 and Supplemental Table 1; supplemental material available online with this article; https://doi.org/10.1172/JCI179391DS1).

All patients exhibited a broad serological response against multiple *Borrelia* antigens; however, the majority did not show detectable responses to OspA (Supplemental Table 1). Some patients carried the well-known ARLA HLA-DRB1 risk alleles *01:01, *04:01, and *15:01, including the only patient with a detectable antibody response against OspA who was homozygous for the *15:01 allele. However, 5 out of the 13 patients (38.5%) carried at least 1 HLA-DRB1*11 allele, which was underrepresented in the North American ARLA cohort (Supplemental Table 1) (8). In the European Cohort, the cumulative frequency of the group of known HLA-DRB1 “risk alleles” tended to be lower in comparison with the reported North American cohort (23.1% versus 38.1%, *P* = 0.18), whereas the frequency of “protective alleles” was significantly higher (42.5% versus 11.9%, *P* < 0.01; Supplemental Table 2). Thus, despite the similarity in HLA-DRB1 allele distribution among geographically and ethnically matched control cohorts (Supplemental Table 2), these allele groups exhibited contrasting patterns between the 2 patient cohorts.

**Figure 1:**
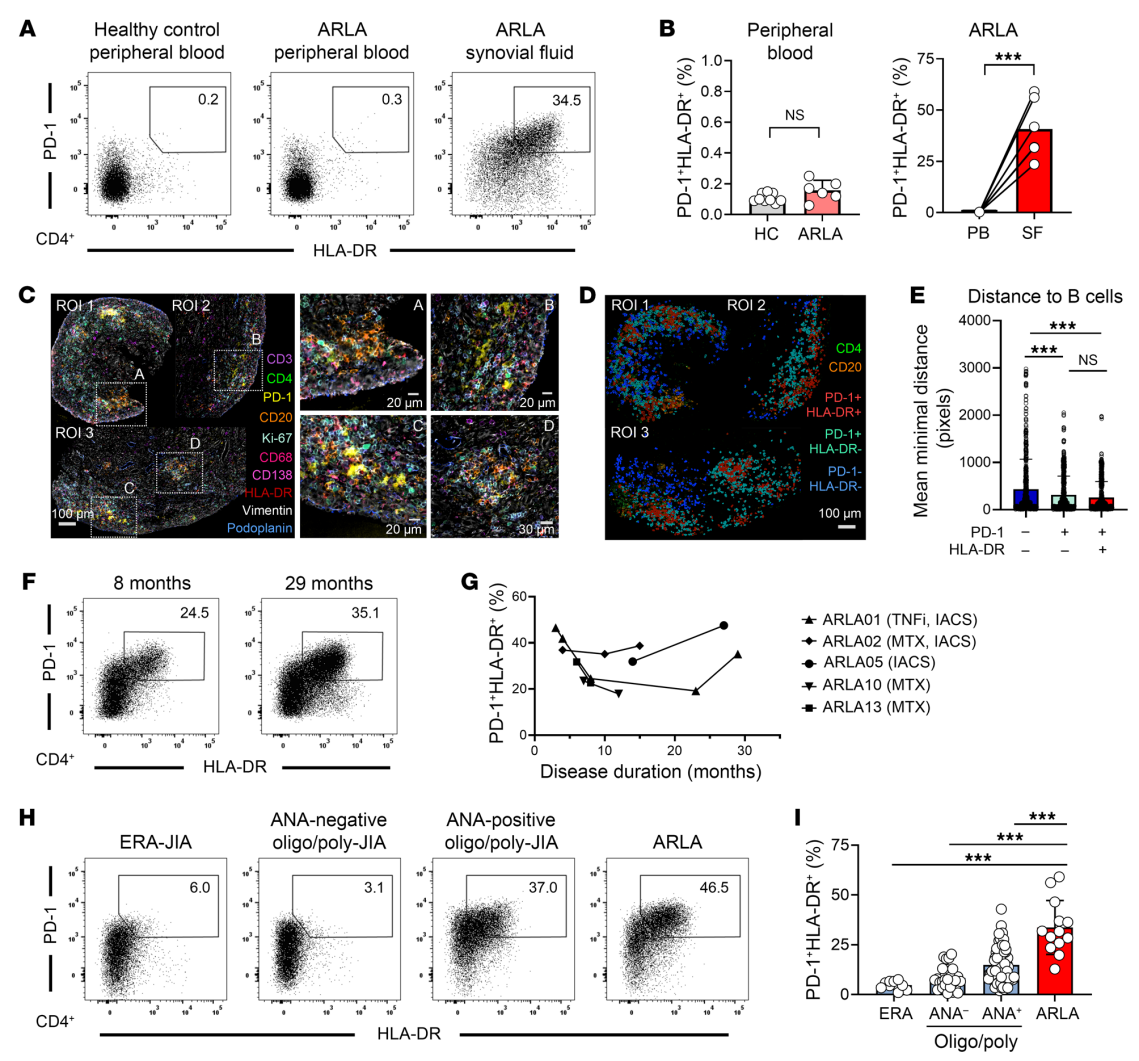
Expansion of PD-1^hi^HLA-DR^+^CD4^+^ effector T cells in the joints of patients with ARLA throughout disease course. (**A**) Representative dot plots showing PD-1 and HLA-DR expression on peripheral blood CD4^+^ T cells from healthy controls and matched peripheral blood and SF CD4^+^ T cells from patient ARLA05. (**B**) Compiled data from 9 people in the healthy control (HC) group and 6 patients with ARLA indicating PD-1^hi^HLA-DR^+^ T cell frequencies in peripheral blood (PB) and matched SF. Bars represent mean frequency ± SD. Unpaired (left) and paired (right) 2-tailed Student’s *t* test, ***P < 0.001. (**C**) Immunofluorescence images using MICS technology of 3 regions of interest (ROI) of synovial tissue sections from ARLA01 with indicated stain markers. Scale bars: 100 μm (left image); 20 μm (2 top right images and 1 bottom left); 30 μm (bottom right image). (**D**) Spatial mapping of 3 CD4^+^ T cell subsets gated based on their PD1 and HLA-DR expression and projected on segmented data of ROIs. Scale bar: 100 μm (**E**) Average minimum distance of each segmented CD20^+^ B cell to segmented CD4^+^ T cell subsets. 1-way ANOVA with Tukey’s multiple comparisons test; ***P < 0.001. (**F**) Dot plots showing PD-1 and HLA-DR expression on SF CD4^+^ T cells from patient ARLA02 at different time points. (**G**) Distribution of SF PD-1^hi^HLA-DR^+^CD4^+^ T cell frequencies in 5 ARLA patients observed throughout the disease course; intraarticular corticosteroids (IACS), TNF-α inhibitor (TNFi), methotrexate (MTX). (**H**) Dot plots demonstrating PD-1 and HLA-DR expression on SF CD4^+^ T cells from patients with JIA and ARLA. (**I**) Distribution of SF PD-1^hi^HLA-DR^+^CD4^+^ T cell frequencies stratified based on disease subgroup and/or antinuclear antibody (ANA) status of patients with JIA and ARLA. ERA, enthesitis-related arthritis. Bars indicate mean frequency ± SD; Dunnett’s multiple comparisons test from ordinary 1-way ANOVA, ***P < 0.001.

Effector T cells present an instructive cell subset for investigating ongoing immune responses, as they arise from the division of antigen-specific T cells upon TCR activation and are enriched with antigen-specific clones. Recent evidence suggests that disease-specific effector Th cells can be distinguished based on the expression of distinct activation markers (23). To analyze the distribution of recently antigen-activated effector CD4^+^ T cells in the inflamed joints of patients with ARLA, we utilized flow cytometry and employed high expression levels of PD-1 and HLA-DR as surrogate markers. A significant proportion of PD-1^hi^HLA-DR^+^CD4^+^ T cells was observed in the SF but not in the peripheral blood of patients with ARLA, and these cells exhibited enrichment in CD45RO^+^CCR7^–^ effector memory phenotype (Figure 1A, and B, and Supplemental Figure 2A). Analyzing the clonal diversity by sequencing the TCR-β repertoire within bulk-sorted SF PD-1^hi^HLA-DR^+^ and PD-1^lo^HLA-DR^–^ CD4^+^ T cells revealed a more restricted repertoire in the PD-1^hi^HLA-DR^+^ subset as an indicator of oligoclonal expansion (Supplemental Figure 2B). Additionally, the PD-1^hi^HLA-DR^+^CD4 T cell subset demonstrated a significant enrichment in cells expressing Ki-67 as sign of ongoing proliferation (Supplemental Figure 2, C and D).

PD1^hi^HLA-DR^+^CD4^+^ T cells were observed in comparable frequencies in both SF and matched synovial tissue samples, with a substantial clonal overlap evident between these compartments (Supplemental Figure 3, A–C). IHC analysis revealed synovial hyperplasia and synovial infiltration of PD-1–expressing CD4^+^ T cells as well as CD20^+^ B cells (Supplemental Figure 3D). To track PD1^hi^HLA-DR^+^CD4^+^ T cells in their spatial context at single-cell resolution, we employed MACSima Imaging Cycling Staining (MICS) to analyze 3 distinct regions of interest (ROI) across 1 tissue section from a representative patient (ARLA01). We observed a dense infiltrate of CD4^+^ T cells dispersed across the synovial sublining layer (Figure 1C). Within this infiltrate, few scattered CD20^+^ B cells were present, while CD138^+^ plasma cells were located in the synovial stroma. Additionally, several lymphoid aggregates were identified, comprised of CD4^+^ T cells with elevated PD-1 expression and CD20^+^ B cells (Figure 1C). Next, we quantified the spatial distribution of CD4^+^ T cells based on the expression levels of PD-1 and HLA-DR in segmented cells. PD-1^–^HLA-DR^–^ cells were loosely scattered, whereas PD-1^+^HLA-DR^+^ were localized at the centers of the lymphoid aggregates and surrounded by PD-1^+^HLA-DR^–^CD4^+^ T cells (Figure 1D). Among these 3 CD4^+^ T cell subsets, PD1^+^HLADR^+^ and PD-1^+^HLA-DR^–^ cells exhibited shorter average minimum distance to CD20^+^ B cells compared with PD-1^–^HLA-DR^–^ cells, suggesting ongoing T/B cell interaction in theses lymphoid aggregates (Figure 1E).

We were able to track the expansion of PD-1^hi^HLA-DR^+^CD4^+^ T cells during the disease course in 5 patients with ARLA. Remarkably, a high frequency of PD-1^hi^HLA-DR^+^CD4^+^ T cells persisted in their joints for up to 2.5 years after the onset of arthritis, correlating with ongoing synovitis despite prior antibiotic treatment and concurrent antiinflammatory medication (Figure 1, F and G, and Supplemental Figure 1). The frequencies of PD-1^hi^HLA-DR^+^CD4^+^ T cells in the SF of patients with ARLA were found to be significantly higher compared with age-matched patients with various subtypes of juvenile idiopathic arthritis (JIA), which served as disease controls (Figure 1, H and I). Notably, the frequency of PD-1^hi^HLA-DR^+^CD4^+^ T cells in ARLA SF even exceeded that observed in patients with antinuclear antibody–positive JIA, a condition in which a local autoimmune response is suggested to drive T effector cell expansion (24).

Collectively, our data characterize the PD-1^+^HLA-DR^+^ CD4^+^ T cell subset in the joints of patients with ARLA as oligoclonally expanded effector cells that may localize within lymphoid aggregates within the synovia. These findings underscore their role as locally induced effector cells and highlight their potential utility in dissecting the pathogenic T cell response within the inflamed joints of patients with ARLA.

### The TCR repertoire of PD-1^hi^HLA-DR^+^ CD4^+^ T cells in the joints of patients with ARLA displays signs of an ongoing and convergent T cell response.

**Figure 2:**
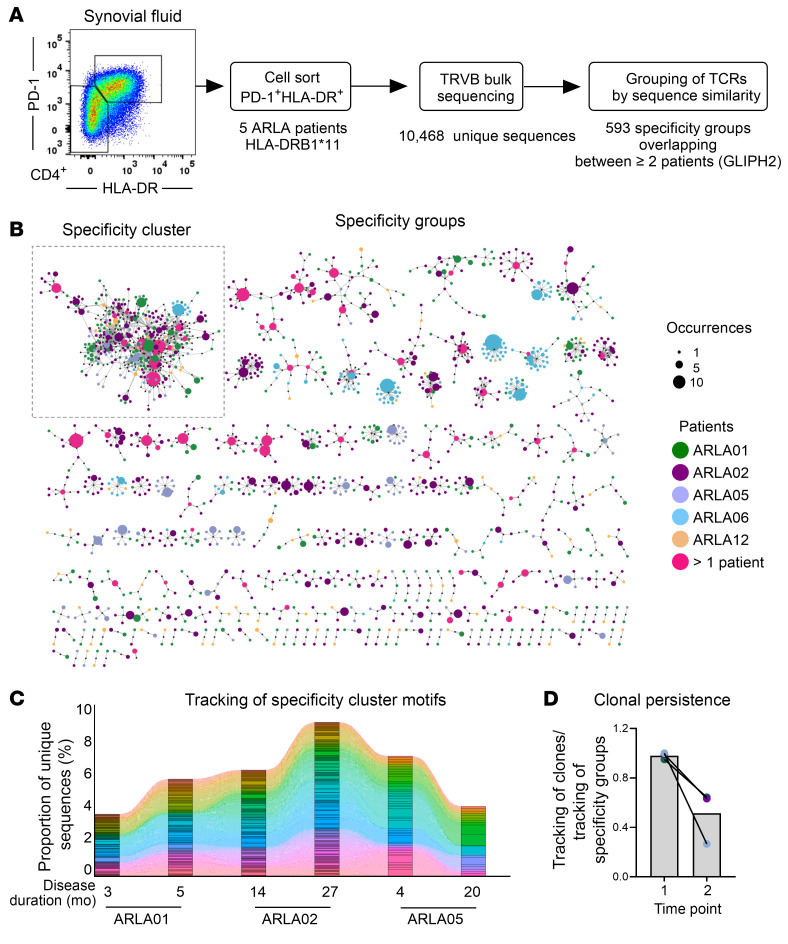
Convergent and ongoing T cell responses in the joints of patients with ARLA. (**A**) Schematic work-flow illustrating the analysis process for identification of TCR similarities and clustering of TCRs into groups based on their probable specificity. (**B**) Network representation displaying TCR specificity groups enriched by GLIPH2 in SF PD-1^hi^HLA-DR^+^CD4^+^ T cells from 5 patients with ARLA with at least 1 HLA-DRB1*11 allele. Only specificity groups containing sequences from multiple patients are shown. Motifs are represented by small black circles, and corresponding CDR3-β sequences as colored circles; colors correspond to the sourcing individual sizes indicate the absolute abundancies of unique CDR3 amino acid (aa) sequences in all patients. (**C**) Tracking of occupied repertoire space within SF PD-1^hi^HLA-DR^+^CD4^+^ T cells using sequences containing CDR3 aa motifs from the specificity cluster at various time points in 3 patients. Each color corresponds to an unique CDR3 aa sequence. (**D**) Ratio comparison of the occupied repertoire space by sequences containing CDR3 aa motifs from the specificity cluster, defined in **B**, against the occupied repertoire space by CDR3 aa sequences in the ‘specificity cluster’ at time point 1 (as depicted in Supplemental Figure 2A).

We speculated whether the sustained expansion of PD-1^hi^HLA-DR^+^CD4^+^ T cells in patients with ARLA throughout the disease course might be driven by persistent recognition of disease-specific antigens. To investigate this, we first analyzed the TCR repertoire of these cells for indicative markers. Considering HLA restriction and the unexpectedly high frequency of the HLA-DRB1*11 allele in our ARLA cohort, we initially focused our analysis on patients who carried at least 1 HLA-DRB1*11 allele (Supplemental Tables 1 and 3). To comprehensively assess the TCR repertoire on a larger scale, we sorted SF PD-1^hi^HLA-DR^+^CD4^+^ T cells from 5 patients and analyzed their TCR-β repertoire by bulk sequencing. Each sample yielded between 529 and 4,774 distinct clones, with minimal clonal overlap observed among the analyzed individuals (0, 0, or 5 shared clonotypes between 5, 4, or 3 individuals, respectively). As this approach failed to reveal significant numbers of shared clonotypes indicative of a disease-specific TCR response, we next utilized GLIPH2 (Grouping of Lymphocyte Interactions by Paratope Hotspots, version 2) (25). This algorithm clusters TCRs based on shared sequence similarities rather than identities, predicting them to bind the same MHC-restricted peptide antigen. By applying this method to the combined set of bulk TCR-β sequences, we identified 593 different specificity groups overlapping between at least 2 individuals (Figure 2A). Performing a network visualization of those sequences and their specificity groups, we identified a cluster of significantly enriched TCRs that were closely linked by similar specificity groups (highlighted as ‘Specificity Cluster’ in Figure 2B). This cluster comprised 3.0% to 6.7% of the total clonal space and was enriched with specificity groups that shared similar local motifs located in the part of the CDR3-β not coded for by germline *TRBV* and *TRBJ* segments (therefore n–/p– nucleotides, Table 1).

We next sought to investigate the longitudinal kinetics of the specificity cluster within the PD-1^hi^HLA-DR^+^ cell subset in SF samples from 3 patients with ARLA collected at various time points during disease course. TCRs carrying specificity cluster motifs remained detectable in subsequent samples at frequencies comparable to those observed during the initial sampling, with clonal persistence even after intervals of up to 16 months between analysis time points (Figure 2C and Supplemental Figure 4A). In addition, nearly half of the TCRs associated with any specificity cluster motifs in the follow-up samples could not be identified in the initial sample, suggesting that new clones with similar TCRs are recruited into the specificity cluster over time (Figure 2D and Supplemental Figure 4A). Exploring deeper into the CDR3-β sequences corresponding with the most frequent specificity groups in the cluster, we uncovered greater nucleotide diversity compared with the amino acid (aa) level as a characteristic sign of a convergent T cell response (Supplemental Figure 4, B and C).

Thus, within SF PD-1^hi^HLA-DR^+^CD4^+^ T cells of patients with ARLA exhibiting a distinct HLA-DRB1 background, we identified a persistent cluster of TCR specificity groups that endured throughout the disease course. This cluster was partially replenished over time by different clones with identical TCR motifs. This observation aligns with a continually ongoing T cell response in the joints of these patients with ARLA, triggered by and converging toward a set of antigens.

### A HLA-DRB1–restricted TCR-β amino acid motif functions as surrogate marker for ARLA-specific TCRs.

To facilitate the identification of disease-specific TCRs in ARLA, our objective was to uncover surrogate markers capable of comprehensively identifying these TCRs with minimal effort. To achieve this, we conducted a detailed analysis of the fundamental molecular patterns exhibited by the TCRs within the previously defined specificity cluster.

**Figure 3:**
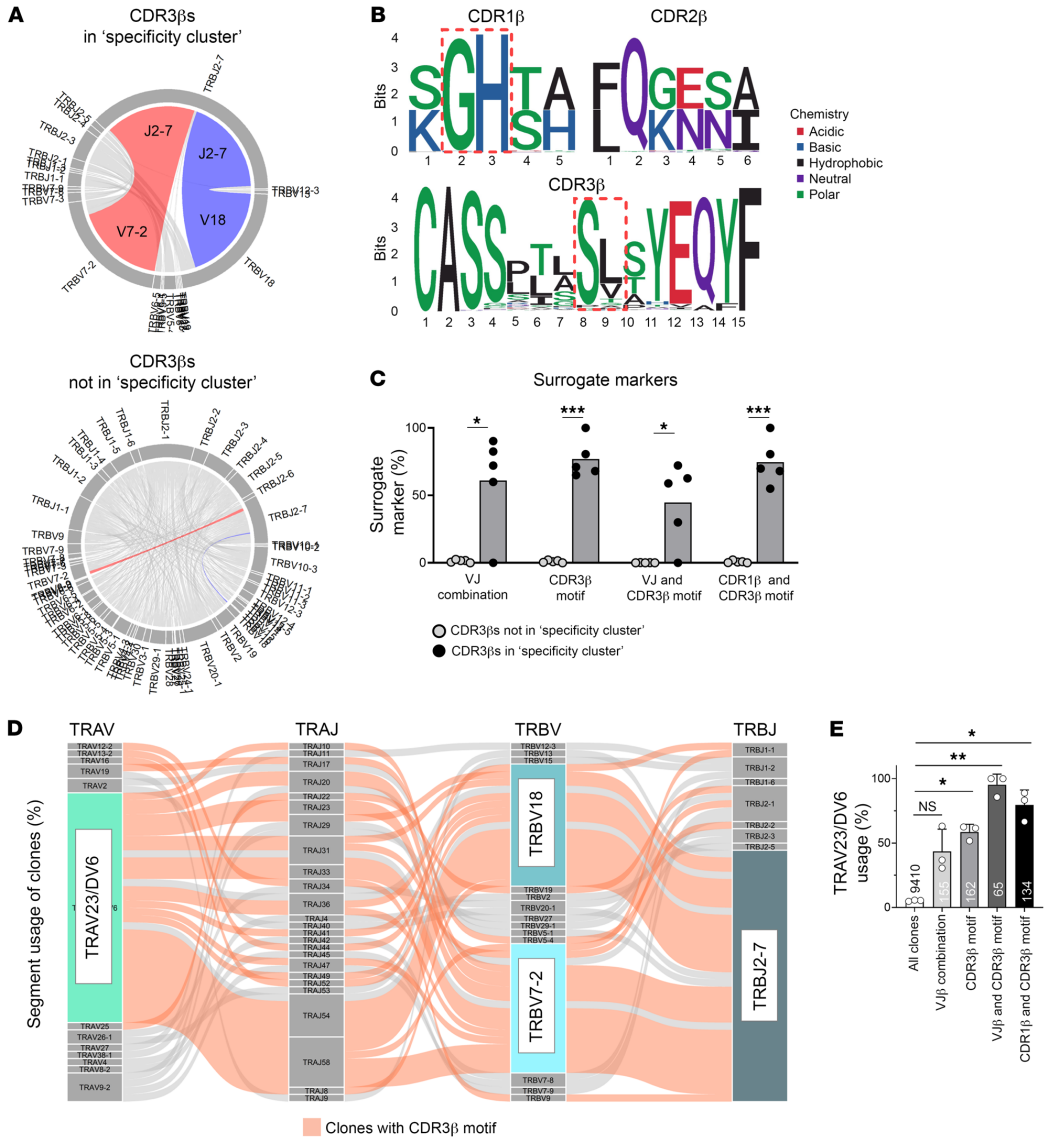
A combined CDR1β / CDR3β surrogate marker defines a common disease-associated TCRs motif. (**A**) The distribution of TRBV-TRBJ gene segment pairings is depicted in circos plots, showing unique TCRβ chain sequences derived from SF PD-1^hi^HLA-DR^+^CD4^+^ T cells collected from 5 ARLA patients. The upper circle delineates sequences belonging to the ‘specificity cluster,’ as illustrated in Figure 2A, while the lower circle represents the remaining sequences. The TRBV7-2.TRBJ2-7 and TRBV18.TRBJ2-7 pairing are highlighted in red and blue, respectively. (**B**) Sequence plots depicting the amino acid sequences in CDR1-3β derived from sequences within the specificity cluster. For the generation of sequence plots, TCR sequences were filtered to include the most abundant length of each CDR. Potential surrogate markers, such as GH in CDR1-β (CDR-1β motif) and SL/SV in CDR3-β (CDR3β motif), are outlined in red. (**C**) The frequencies of the indicated surrogate markers are compared between sequences within and outside the specificity cluster; *P* values determined by multiple paired *t* tests are adjusted for multiple testing by Holm-Šídák method; *P < 0.05, ***P < 0.001 Bars indicate mean ± SD. (**D**) Alluvial plot of TRAV-TRAJ-TRBV-TRBJ combinations (determined by paired TCR-α/β–sequencing of CD4^+^ SF T cells from 3 patients with ARLA) in unique clones containing motifs from the specificity cluster in the CDR3-β. Alluvials from clones with the CDR3-β motif (SL/SV at IMGT position 111/112 in CDR3-β) are highlighted in coral. (**E**) Frequency of clones with TRAV23/DV6 gene segment usage within all clones or within subsets filtered for the indicated properties of the TCR-β chain. The number of clones in each subset is indicated at each bar. Circles represent individual patients, error bars indicate SD. Significances were calculated by 1-way ANOVA and multiple comparisons (to all clones) corrected with Dunnetts formula; *P < 0.05, **P < 0.01.

Analysis of the TCR-β VJ pairings revealed a significant increase of the TRBV7-2.TRBJ2-7 and TRBV18.TRBJ2-7 combinations in TCRs within the cluster compared with all others (74.8% versus 1.5%, respectively; *P* < 0.0001 by χ^2^ with Yate’s correction; Figure 3A). In addition, TCR-β sequences contributed by patient ARLA06 to the specificity cluster displayed a distinct VJ pairing with predominance of TRBV5-4.J2-3 (Supplemental Figure 5A). The TCR-β sequences from all patients in the cluster almost exclusively used the aa doublet ‘SL’ or ‘SV’ within the hypervariable part of the CDR3 region at IMGT position 111 and 112, which were not encoded by germline template and displayed high variability on the nucleotide level (Table 1, Figure 3B, Supplemental Figure 4, B and C, and Supplemental Figure 5B). Additionally, the TCR-β sequences were characterized by usage of ‘GH’ at IMGT position 28 and 29 within the CDR1 region, which reflects the use of the above mentioned Vβ segments 7–2, 18, and 5–4 that inherit this motif in their germline configuration (Figure 3B). The simple CDR3-β motif (‘SV or SL’) — alone or in combination with the CDR1-β motif (‘GH’) — could, within the 5 analyzed patients, identify between 55%–100% of all TCRs that belonged to the cluster (Figure 3C). Notably, the frequencies of these markers also remained unchanged in SF PD-1^hi^HLA-DR^+^CD4^+^ T cells of patients with ARLA during the disease course and were found at similar frequencies in matched synovial tissue (Supplemental Figure 6).

We then aimed at including the TCR-α chain into our analysis. To obtain paired TCR-α/β sequences we performed scRNA-Seq of SF CD4^+^ T cells from 3 patients (Supplemental Table 3). Clones that could be linked to the specificity cluster by TCR-β specificity groups revealed a significant enrichment toward usage of the gene segment TRAV23/DV6 compared with all other clones, which rather displayed random usage of TRAV segments (64% versus 4.7% of the clones respectively, *P* < 0.0001 by Fisher’s exact test; Figure 3D and Supplemental Figure 7A). This significant enrichment of TRAV23/DV6 usage was also observed when filtering the clones for the above delineated surrogate marker combinations of the TCR-β chain (Figure 3E and Supplemental Figure 7, B and C). Notably, whereas the CDR3-β motif alone identified clones using TRAV23/DV6 with a frequency of 52% to 62% among analyzed patients, the combination of the CDR1-β and CDR3-β motifs increased this frequency to 67%–89% without substantial loss of identified clone numbers (Figure 3E). Hence, the combination of the CDR3-β motif (‘SL’ or ‘SV’ at IMGT positions 111 and 112) with the CDR1-β motif (‘GH’ at IMGT positions 28 and 29), hereafter termed the ‘ARLA motif,’ demonstrated the highest specificity without comprising sensitivity as a surrogate marker for identifying T cell clones that make up the identified specificity cluster in patients with ARLA.

To investigate the specificity of the ARLA motif, we explored the CD4^+^ TCR repertoire within various disease conditions for its presence. Initially, we assessed published TCR-β sequences known to target microbial antigens or autoantigens for the defined surrogate markers (*n* = 2,094 from VDJdb (26), *n* = 12 (27)). The distinct ARLA-associated CDR3-β motif was found in a few sequences, however, the specific combination of this motif together with the distinct VJ combination was not detected in any of the sequences (Supplemental Figure 8; CDR1-β motif frequencies could not be assessed due to missing information in databases). The CDR3-β motif was similarly low in the SF CD4^+^ TCR repertoire from 2 patients in the North American cohort with LA and could not be identified in published TCR-β sequences from HLA-DRB1*04:01 restricted OspA_163-174_-specific T cell clones (Supplemental Figure 8B and Supplemental Figure 9) (27, 28). Additionally, the ‘ARLA motif’ was almost absent in the SF CD4^+^ T cell repertoire in patients with JIA and rheumatoid arthritis (RA) (Supplemental Figure 9) (28–30).

**Figure 4:**
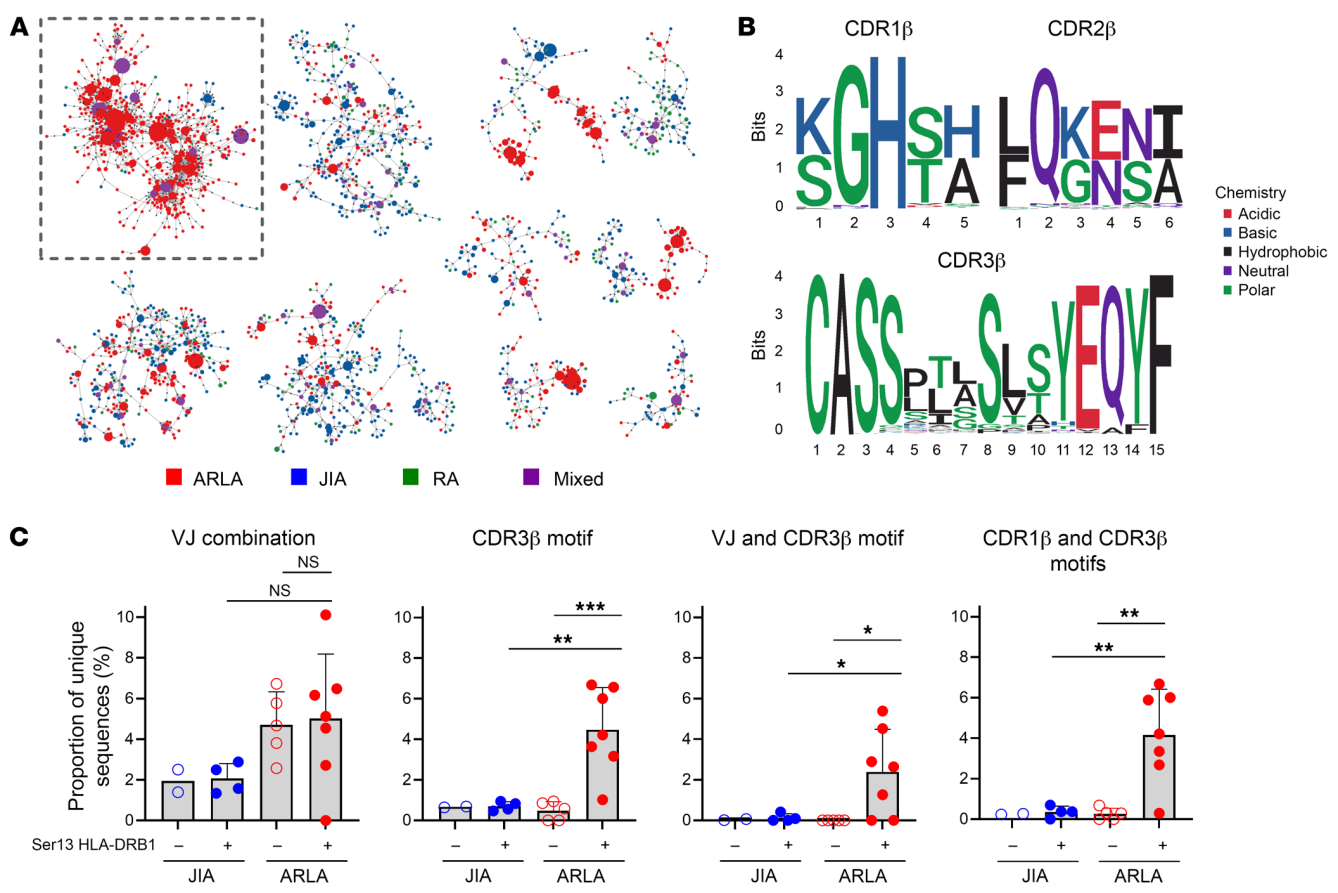
Surrogate markers for ARLA-associated TCRs are disease specific and HLA-DRB1 restricted. (**A**) Network representation of TCR specificity groups enriched by GLIPH2 in SF PD-1^hi^CD4^+^ T cells from 12 patients with ARLA, 6 with JIA and 3 with RA. Only specificity groups containing sequences from multiple patients are included and only networks with at least 50 members are shown. Motifs are represented by small black circles and corresponding CDR3 sequences by colored circles. The circle sizes reflect the absolute abundances of unique CDR3 amino acid (aa) sequences across all patients. (**B**) Sequence plots showcasing the aa sequences in CDR1-3β, derived from sequences within the highlighted network on the left, are displayed. To generate these sequence plots, sequences were filtered for the most abundant length of each CDR. (**C**) Frequencies of indicated surrogate markers in TCR-β sequences of FACS-sorted SF PD-1^hi^HLA-DR^+^CD4^+^ cells from children with JIA (*n* = 6) and ARLA (*n* = 12) determined by bulk sequencing. Patients exhibiting Serine at position 13 (Ser13) of HLA-DRB1 on at least 1 allele are denoted by filled circles. Bars indicate mean ± SD. 1-way ANOVA and multiple comparisons (to ARLA +) corrected with Dunnetts formula; the Ser13– JIA group was excluded from the statistical analysis due to the small sample size of *n* = 2; NS: *P* > 0.05, *P < 0.05, **P < 0.01, ***P < 0.001.

We next extended our analysis from the 5 patients with at least 1 HLA-DRB1*11 allele to all 12 patients with ARLA with available TCR sequences and performed GLIPH2 on TCR-β sequences derived from bulk sorted PD-1^hi^CD4^+^ T cells from all patients with ARLA as well as on published data from patients with RA or JIA (input: 20,486, 25,095, and 8,698 sequences from ARLA, JIA, and RA respectively (24, 29)) and visualized the results using network analysis. Remarkably, a cluster was identified almost exclusively composed of sequences from ARLA cells (93.6% of TCR sequences from ARLA, 96.4% of those from patients with ARLA with HLA-DRB1*11), with no such private clusters existing for the other 2 diseases (Figure 4A). The majority of clones in this cluster exhibited TCR-β sequences matching the ARLA motif (Figure 4B). To consider the patients’ genetic background, we compared TCR-β repertoires from SF PD-1^hi^HLA-DR^+^ CD4^+^ T cells of patients with ARLA and JIA with known HLA-DRB1 genotypes. The ARLA motif was only present in patients with ARLA carrying HLA-DRB1*11 and HLA-DRB1*13 alleles. These alleles could be distinguished from all other HLA-DRB1 alleles present in the cohort by the presence of serine at position 13 (Ser13), which is known to influence specific peptide binding properties and shape TCR selection (Supplemental Figure 10) (31, 32). The patient groups did not differ in the general distribution of the distinct TCR-β V or J genes used by the ARLA motif (Supplemental Figure 11). However, the distinct ARLA motif was significantly enriched in HLA-DRB1 Ser13 positive patients with ARLA compared with patients with ARLA without Ser13 alleles and patients with JIA carrying a Ser13 allele (Figure 4C). This observation could be replicated in another data set focusing on total CD4^+^ T cells derived from SF of patients with JIA and ARLA (Supplemental Figure 12) (28). Hence, focusing on patients with ARLA with a distinct HLA-DRB1 background allowed us to identify a TCR-β motif that served as a surrogate marker for the identification of ARLA-specific TCRs.

### Clonally expanded peripheral T helper cells dominate the CD4^+^ T cell landscape in the joints of patients with ARLA.

To link TCR specificity to function, we next aimed to analyze the phenotype of CD4^+^ T cells expressing ARLA-specific TCRs in more detail. For this approach, we first comprehensively characterized the phenotype, functional state, and clonality of SF Th cells in ARLA by conducting an in-depth investigation using combined scRNA-Seq and single-cell TCR sequencing (scTCR-Seq; 10X Genomics) of sorted CD4^+^ T cells obtained from the SF of 3 patients with ARLA (Supplemental Table 3). Following demultiplexing, quality control, and data integration, we successfully identified and analyzed a total of 12,622 CD4^+^ T cells for transcriptomic analysis.

**Figure 5:**
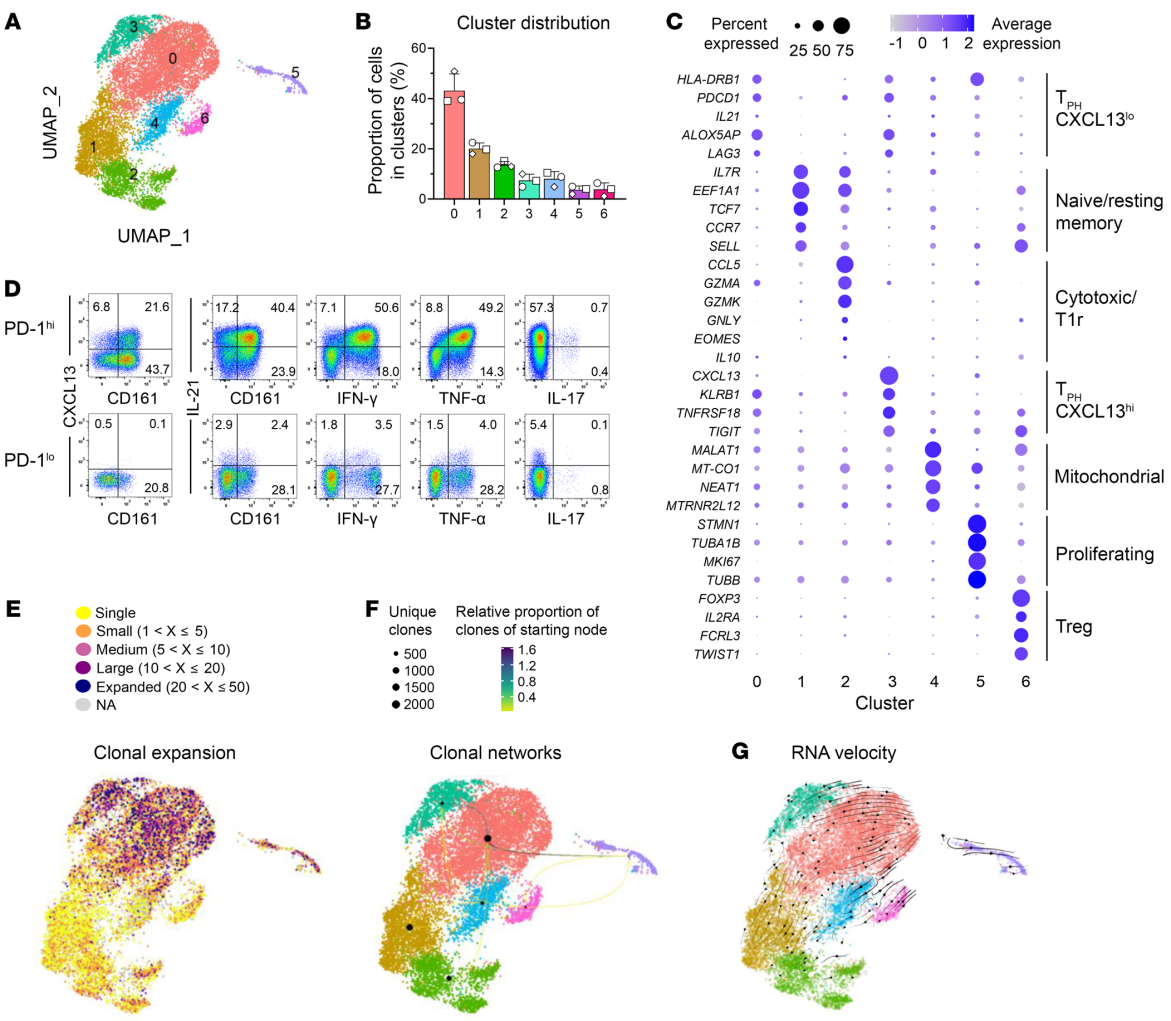
The synovial CD4^+^ T cell landscape in ARLA is dominated by clonally expanded T_PH_ cells. (**A**) UMAP representations of cell clustering via scRNA-Seq analysis of SF CD4^+^ T cells from 3 patients with ARLA. (**B**) Mean frequency of cells allocated to the respective clusters. Symbols represent individual patients, error bars indicate SD. (**C**) 2-D dot plot showing the expression of selected marker genes. The area of the dots indicates the percentage of cells within the cluster expressing the gene, the color represents the average expression level. (**D**) Representative dot blots showing cytokine expression in SF PD-1^hi^ (upper row) and PD-1^lo^ CD4^+^ cells, assessed by flow cytometry. (**E**) Clonal expansion is depicted based on the size of individual clones, determined through paired TCR-α/β sequencing, represented on the UMAP plot from **A**. (**F**) Clonal connectivities between individual clusters are illustrated by arrows, with darker colors indicating a higher proportion of clones originating from the starting cluster. (**G**) Possible developmental trajectories projected onto the UMAP representation from **A**, inferred by RNA velocity analysis.

By performing clustering analysis on the integrated transcriptomic data, we identified 7 distinct clusters within the SF CD4^+^ T cells (Figure 5A). The cluster were equally represented between the 3 patients and TCR-Seq revealed sufficient coverage (Supplemental Figure 13). Cluster 0 emerged as the dominant cluster, exhibiting transcriptional similarities with cluster 3. Remarkably, these 2 clusters constituted approximately half of the total T cells in each patient (Figure 5B). Both clusters displayed heightened expression of *HLA-DR*, *PDCD1*, (encoding PD-1), and *IL21* (encoding IL-21). Additionally, we observed upregulation of *LAG3*, *ALOX5AP*, and *KLRB1* (encoding CD161) in both clusters, which, together, are well-established transcriptomic markers of peripheral T helper (T_PH_) cells (Figure 5C, Supplemental Figure 14 and Supplemental Table 4). Despite their similarities, only a few genes showed differential expression between the 2 T_PH_ cell clusters, with *CXCL13* being the most significant (Supplemental Table 5). This finding led us to classify cluster 0 and cluster 3 as CXCL13^lo^ T_PH_ cells and CXCL13^hi^ T_PH_ cells, respectively. The characteristic chemokine/cytokine pattern of the T_PH_ cluster with expression of CXCL13, IL-21, and IFN-γ could be recapitulated on protein level using flow cytometry (Figure 5D). Cluster 1 exhibited a significant enrichment in the expression of naive T cell markers (*CCR7*, *SELL*, and *TCF7*) as well as central memory marker genes (*IL7R* and *EEF1A1*) resulting in classification of cluster 1 as a naive/resting memory T cell cluster. In contrast, cluster 2 showed heightened expression of cytotoxic marker genes but also immune regulatory genes (*IL10*), while cluster 6 displayed an abundance of marker genes associated with regulatory T cells (Treg) (Figure 5C, Supplemental Figure 14 and Supplemental Table 4). As a result of these distinctive expression profiles, we labeled cluster 2 as a cytotoxic/T regulatory 1 (Tr1) cluster, and cluster 6 as a Treg cluster. Cluster 4 exhibited differential expression of mitochondrial genes and genes previously reported to be expressed in Humanin and SESN3 CD4^+^ T cells in the SF of patients with RA (33). Finally, the remaining cells in cluster 5 were predominantly characterized by marker genes associated with proliferating CD4^+^ T cells, indicating an actively dividing subpopulation within the synovial T cell pool (Figure 5C, Supplemental Figure 14 and Supplemental Table 4).

To gain further insights into the tissue-specific clonal expansion and activation of each T cell cluster, we conducted an analysis of the TCR usage among all T cells that exhibited a productive TCR-β sequence. By integrating the scTCR-Seq data into the analysis, we observed the most significant clonal expansion within the 2 T_PH_ clusters (Figure 5E and Supplemental Figure 15A). Notably, the largest clonal overlap was observed between these 2 T_PH_ clusters and the cluster of proliferating cells (Figure 5F and Supplemental Figure 15B), suggesting a potential shared developmental trajectory among these 3 clusters. In contrast, the overlap between the T_PH_ clusters and the 2 regulatory clusters (Tr1, cluster 2 and Treg, cluster 6) was minimal. RNA velocity analysis supported this observation and could not reveal a trajectory between the T_PH_ clusters and the other effector T cell clusters (Figure 5G). In conclusion, these findings characterize SF T_PH_ cells as the dominant clonally expanding cell population, suggesting that these cell subsets might reflect the ongoing T helper response in the inflamed joints of patients with ARLA.

### T cells with ARLA specific TCRs reside in T_PH_ clusters and show signs of TCR-driven activation.

Having identified surrogate markers for disease-specific TCRs in patients with ARLA, we finally aimed at connecting TCR specificity with T cell phenotype and function. To achieve this, we mined the combined scRNA-Seq/scTCR-Seq data set of the 3 patients with ARLA (each of them carrying at least 1 HLA-DRB1*11 allele) for T cell clones that displayed the TCR-β ARLA motif (CDR1-β and CDR3-β). Out of 9,344 total cells, we identified 158 cells harboring this motif. To distinguish cells with TCR specificities unrelated to ARLA and potentially activated by bystander activation, we used TCR-β sequences with known specificities against viral antigens independent of a matching HLA background from the public VDJdb database to infer viral motifs through the GLIPH2 algorithm (Supplemental Table 6) (26). We then searched the scRNA-Seq/scTCR-Seq data set from patients with ARLA for these viral motifs, resulting in a group of 625 cells.

**Figure 6:**
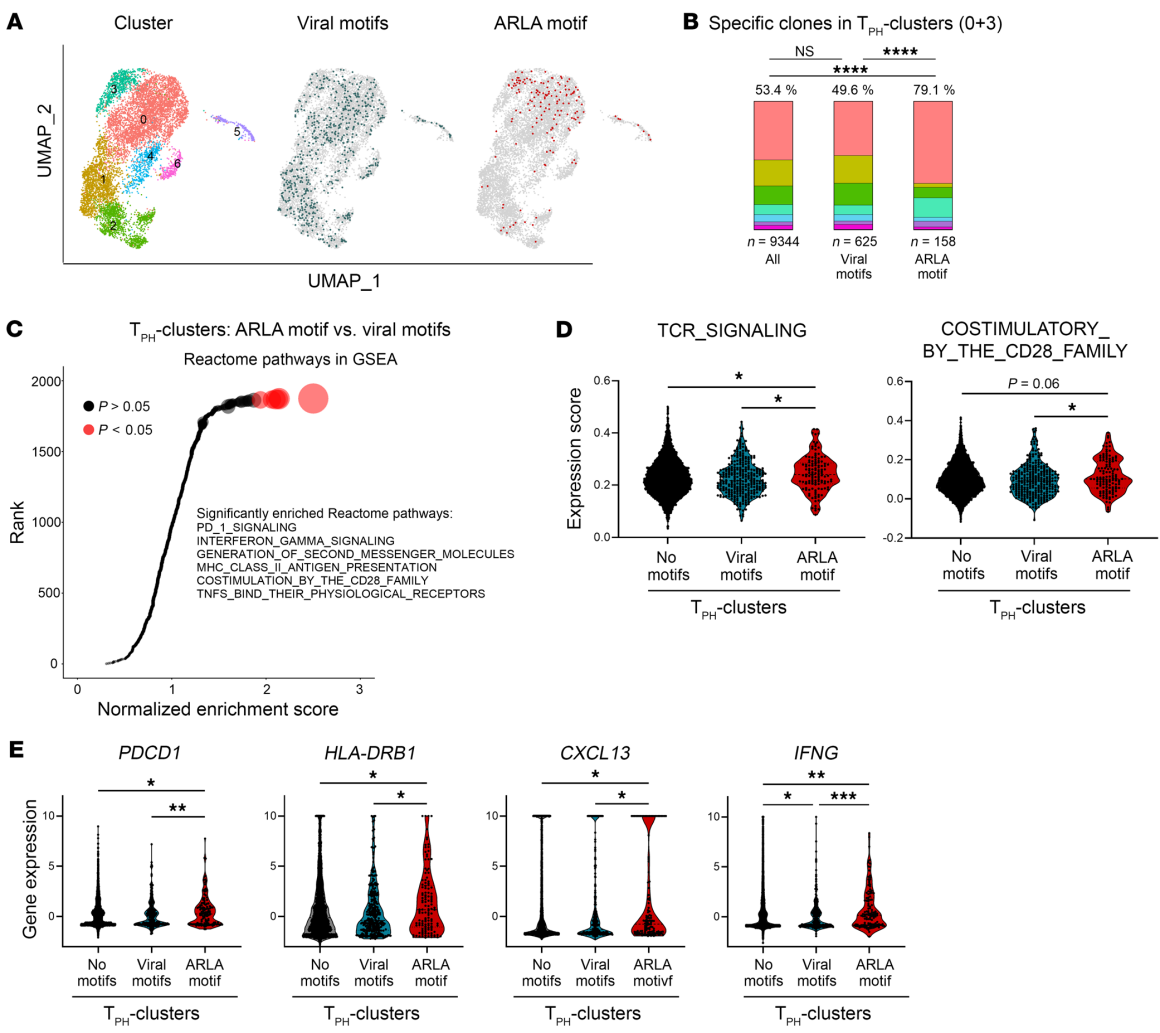
ARLA-specific T cell clones map to the T_PH_ cluster and show signs of TCR-driven activation. (**A**) UMAP representations of cell clustering via scRNA-Seq analysis of SF CD4^+^ T cells from 3 ARLA patients (from Figure 5A). In the center, cells with viral TCR motifs are highlighted, while on the right those with the ARLA motifs (CDR1-β and CDR3-β motifs) are highlighted. (**B**) Relative distribution of cells within each cluster, categorized by color as depicted in **A**, considering all cells or cells containing either viral or ARLA TCR motifs. Fisher’s exact test between indicated groups; ****P < 0.0001. (**C**) Normalized enrichment scores (NES) from Gene Set Enrichment Analysis (GSEA) analysis using Reactome pathways with differentially expressed genes between T_PH_ cells (cluster 0 and 3) with either viral or ARLA TCR motifs. Pathways are ranked based on their NES, with circle size corresponding to negative log_10_ (adjusted *P* value). Red circles represent pathways with an adjusted *P* value < 0.05. The significantly enriched pathways are identified by name. (**D**) Genes associated with TCR signaling-related GSEA pathways were used as input for the AddModuleScore function from Seurat. Resulting scores per cell are plotted and compared between T_PH_ cells containing no motifs, viral or ARLA TCR motifs. (**E**) Expression of selected activation and effector genes in the same groups as in **D**. 1-way ANOVA with Tukey’s multiple comparisons test; *P* values < 0.1 are shown, *P < 0.05, **P < 0.01, ***P < 0.001.

Our analysis applying these surrogate markers revealed that the majority of cells with TCRs containing the ARLA motif resided in the T_PH_ cell clusters 0 and 3 and marginally in proliferating cluster 5 (Figure 6, A and B). However, they were almost absent in the Treg cluster 6 as well as in the Tr1 cluster 2. Additionally, when assessing the relative enrichment of the most frequent aa doublets (more than 1% of doublets) at certain positions in the CDR3-β in an unbiased manner, the only enrichment above background was observed for the doublet CDR3-β 111/112, which constitutes the ARLA CDR3-β motif, in the T_PH_ clusters and proliferating cluster (Supplemental Figure 16). In contrast, T cells expressing TCRs with viral motifs were randomly distributed across the 7 clusters, resembling the distribution of T cells not assigned to either TCR motif group (Figure 6, A and B). Consistent with this observation, the proportion of cells in expanding clones was significantly higher in the ARLA group, and, among them, the frequency of convergent clones appeared to be higher in cells with ARLA motif (Supplemental Figure 17, A and B).

Subsequently, we investigated the transcriptome of ARLA-specific clones to understand their modes of cellular activation. To reduce a bias by cells with viral TCR motifs from non-T_PH_ clusters, we exclusively focused the analysis on cells from the T_PH_ clusters (0 and 3). By applying differentially expressed genes between T_PH_ cells with ARLA motifs and viral motifs to GSEA Reactome pathway analysis, we found a significantly increased normalized enrichment score for pathways indicating signaling via the TCR and costimulatory receptors in the ARLA motif group (Figure 6C). Additionally, creating scores applicable to single-cell gene expression data by utilizing genes from GSEA pathways for TCR signaling and costimulatory signaling resulted in significantly higher scores for T_PH_ cells in the ARLA motif group compared with both the group of T_PH_ cells with viral motifs and that of T_PH_ cells lacking any of these motifs (Figure 6D). Hence, even within the T_PH_ clusters those T cells expressing ARLA-specific TCR motifs showed increased signs of continuous antigen engagement.

Finally, we assessed the effector programs of T_PH_ cells with ARLA-specific TCRs by comparing the expression of selected effector genes within these cells to T_PH_ cells expressing TCRs with no or viral motifs. Despite using exclusively T_PH_ cells as controls, genes associated with the T_PH_ phenotype (*PDCD1* and *HLA-DRB1*) and function (*CXCL13*) showed even higher expression in the group with ARLA motifs (Figure 6E). Additionally, T_PH_ cells bearing the TCR ARLA motif also displayed higher expression of *IFNG,* indicating an upregulated proinflammatory effector program in these cells (Figure 6E). In conclusion, within the inflamed joints of patients with ARLA, we could track T cells with disease-specific TCRs that display signs of ongoing TCR triggering and express a pathogenic effector program resembling T_PH_ cells.

## Discussion

By employing high-throughput TCR sequencing in conjunction with scRNA-Seq, we have identified surrogate markers associated with disease-specific TCRs in postinfectious LA. These markers can be harnessed to dissect the pathogenic T cell response in the inflamed joints of patients. Our findings provide evidence that disease-specific T cells are the driving force behind the expansion of a dominant T_PH_ cell population within the joints of patients with ARLA. These cells exhibit sustained proliferation and replenishment throughout the course of the disease, likely in response to antigen-driven TCR signaling. Notably, ARLA-specific T_PH_ cells display a pathogenic effector program with heightened expression of IFN-γ and CXCL13, which have dominant roles in tissue inflammation and tertiary lymphoid structure formation.

Thus far, presumed targets of the pathological T cell response in ARLA encompass a wide spectrum of antigens, ranging from *Borrelia* components to autoantigens associated to vascular tissue (ECGF, annexin A2, and apoB-100) or extracellular matrix (fibronectin-1, laminin B2, and collagen) (16–19, 27, 34, 35). These diverse T cell responses have been integrated into a framework indicating that exaggerated immune reactions to *Borrelia* antigens, such as OspA, might disrupt T cell tolerance, triggering epitope spreading from *Borrelia* antigens to autoantigens like extracellular matrix proteins (7). Notably, T cell responses against these autoantigens have been specifically detected in patients with ARLA using T cell activation assays or peptide MHC tetramers. The use of these techniques exhibits exceptional sensitivity in discerning antigen-specific T cells contingent upon the knowledge of disease-relevant HLA alleles and their associated epitopes. As a further limitation, activation assays can induce significant alterations in antigen-reactive cells, leading to a bias in their phenotypic and functional characterization. Given the ambiguity encompassing the dominant direction of T cell responses in postinfectious ARLA outside North American (21, 22), accurately and comprehensively predicting the primary targets of the pathogenic T cell response might also prove challenging when employing candidate antigen approaches. In order to overcome these challenges, we decided to use an unbiased approach and employed the GLIPH2 algorithm for the clustering of TCRs that exhibit recognition of the same epitope predicated by shared structural similarity within the CDR3 (25). This algorithm has gained widespread utilization for the detection of antigen-specific TCRs in instances where information pertaining to the antigens is not readily available (36). Additionally, by combining TCR sequence information with gene expression data we could link TCR specificity to cellular function. By this approach we are able to provide a data set that may facilitate the comprehensive and unbiased identification of target antigens of disease-associated TCRs.

In the synovial CD4^+^ T cell population present in the joints of patients with ARLA, we observed a significant increase in activated effector T cells, characterized by heightened expression of PD-1 and HLA-DR, suggesting recent antigen-induced activation. By leveraging this particular phenotype to enrich antigen-reactive T cells and employing high-throughput sequencing, we were able to identify shared disease-specific TCR motifs among patients, which were HLA-DRB1–restricted. These motifs were primarily determined by a nongermline encoded SV/SL at a conserved position 111/112 within the CDR3-β and the utilization of V segments containing aa GH at IMGT position 28/29 (CDR1-β), along with the usage of TRAV23/DV6 in the TCR-α, without the identification of a specific motif therein. The canonical CDR1/3-β sequence functioned as a reliable surrogate marker for identification of respective TCRs. A similar structural configuration, characterized by the preferential Vβ/Jβ usage, a nongermline encoded CDR3-β motif and biased *TRAV* pairing, was identified in gluten-specific, HLA-restricted CD4^+^ T cell clones in celiac disease (37). Here, the nongermline CDR3-β motif made crucial interactions with the gliadin epitope as well as the HLA-DQ2.5 allele, thereby acting as a lynchpin. In ARLA, the marked diversity in nucleotide level sequences encoding CDR3-β motifs, its persistent correlation with T cells throughout the course of the disease, and the partial replacement of TCR motif-bearing T cells by distinct clonal populations over time collectively align with the concept of a convergent T cell response instigated by local antigens. This suggestion finds further support in the observed HLA restriction predominantly linked to HLA-DRB1*11 and HLA-DRB1*13, presumably dictated by the presence of specific aa at a discrete positions within the peptide binding pocket 4 of the HLA molecule (HLA-DRB1 Ser13) (31). HLA-DRB1 position 13 has a strong association with CDR3-β aa composition, thereby shaping the TCR repertoire and mediating risk for multiple autoimmune diseases (32, 38).

Unexpectedly, our investigation revealed a notably high prevalence of the HLA-DRB1*11 allele within the cohort of patients with ARLA under scrutiny. This particular allele had previously been identified as a potential protective factor against the development of an antibiotic-refractory disease course in a study of patients in North America (8). The cohort analyzed in our research is comprised of patients with ARLA referred to our center from diverse regions, providing a representative snapshot — albeit with a limited number of participants — of the spectrum of affected children and adolescents in the region of Germany. The distribution of *Borrelia* species and strains in this geographical region shows a notable difference compared with North America. In Germany, *B*. *afzelii* constituted approximately 60% of the infected ticks, followed by *B*. *garinii* and *B*. *burgdorferi s.s.*, each accounting for around 10% (39). In contrast, *B*. *burgdorferi s.s.* stands as the prevailing species in North America (1). Despite this marked difference, it is generally believed that *B*. *burgdorferi s.s.* also remains the predominant species in cases of clinically evident LA in Europe (7). This, along with the prevalence of less inflammatory strains of *B*. *burgdorferi s.s.*, might account for the lower occurrence and reduced disease severity of LA in Europe. Nonetheless, *B*. *afzelii* and *B*. *garinii* could each be detected in up to one-third of patients with LA in Europe (40, 41). Hence, it is reasonable to suggest that the cases involving LA in Europe differs not only in the presence of diverse *B*. *burgdorferi s.s.* strains but also in the distribution of *Borrelia* species when juxtaposed with the North American context. Moreover, this disparity might extend to ARLA, since the distribution of *Borrelia* species in a group of European patients with prolonged course of arthritis despite antibiotic treatment was also diverse and not restricted to *B*. *burgdorferi s.s.* (40). Based on these observations and our current findings delineating a unique HLA-DRB1–restricted TCR response, it is plausible that specific *Borrelia* strains in Europe, not necessarily belonging to the *B*. *burgdorferi* s.s. genospecies, could elicit exaggerated immune responses linked to ARLA, akin to what has been documented for the RST1 OspC type A strains of *B*. *burgdorferi* s.s. in North America (9). Features such as an expanded surface lipoproteome, which may elicit excessive inflammation followed by dysregulated immune responses, could serve as a common paradigm of these virulent strains (10). It is therefore reasonable to assume that, in comparison to North America, different immunodominant *Borrelia* peptides and, consequently, different HLA-DRB1 alleles are involved in the pathogenesis of ARLA in Europe. Our elucidation of TCR motifs within the context of HLA-DRB1*11–associated ARLA will facilitate the prospect of discerning putative target antigens linked to the pathogenic T cell response in these afflicted individuals. This will also enable a comparative analysis with the antigens predominantly associated with known HLA-DRB1 risk alleles within North America.

Unsurprisingly, expansion of effector T cells bearing the presumed ARLA-specific TCR motif could not be documented in RA, in which the HLA-DRB1 Ser13 alleles are protective (38). In contrast, HLA-DRB Ser13 alleles were confirmed to predispose to JIA, which phenotypically may resemble ARLA (42). Of note, the TCR-β repertoire of activated effector cells in the joints of HLA-DRB Ser13–positive patients with JIA without serological evidence of prior *Borrelia* infection was distinct from that of patients with ARLA with absence of the TCR ARLA motif. Hence, TCRs bearing the ARLA motif may either recognize *Borrelia* antigens not present in the joint of patients with JIA or, more likely, recognize autoantigens that are not targeted by the T cell response in JIA. Cumulatively, these observations substantiate the hypothesis that the dominate T cell response within the joints of patients with ARLA is private and differs in its dominant antigen targets from other rheumatic diseases.

Our study was limited in its ability to determine whether the TCRs specifically identified in patients with ARLA are also involved in the initiation of the pathogenic T cell response. While we successfully characterized the T cell response throughout the chronic phase of arthritis, we did not have access to samples from the initial disease onset of patients who later developed ARLA. Although these limitations constrain interpretation, the convergent T cell response observed in patients with ARLA during the late disease course would align well with the proposed epitope spreading model (7, 16). The observation that T cells carrying TCRs with known specificities against viral antigens were randomly distributed among various T cell clusters and not enriched in the expanded T_PH_ effector cell cluster also refutes bystander activation as the primary mechanism by which T cells are recruited within the local T cell response.

The synovial transcriptomic landscape in patients with ARLA is characterized by a pronounced IFN-γ expression signature and high frequencies of IFN-γ–secreting T cells are present in SF of these patients (43–45). Further, autoreactive T cells, identified through peptide MHC tetramers recognizing extracellular matrix proteins, are notably enriched in T-bet–expressing cells (16). This observation implies that the pathogenic T cell response in ARLA is mediated by Th-1 cells. Our study refines this assumption by demonstrating that ARLA-specific T cells exhibit elevated levels of IFN-γ expression but can be more accurately classified as T_PH_ cells rather than classical Th-1 cells. T_PH_ cells are pivotal drivers of disease pathogenesis in numerous autoimmune conditions and are expanded in inflamed tissues of patients with RA, JIA, and systemic lupus erythematosus (SLE) (24, 46, 47). The phenotype and function of T_PH_ cells exhibit partial overlap with follicular T helper cells, expressing B cell–supporting cytokines such as IL-21 and CXCL13 (48). However, they rather possess a chemokine receptor profile facilitating their accumulation at inflamed sites, along with Th-1–like features including T-bet, IFN-γ and TNF-α expression. Remarkably, T cells bearing the ARLA motif were almost undetectable within the local Treg population as well as in the Tr1 cluster. These findings reinforce earlier observations of a disturbed Treg compartment in ARLA, indicating a lack of recruitment of T cells with regulatory functions into the local T cell response (49, 50). Our findings establish T_PH_ cells as the dominant population among synovial T helper cells in patients with ARLA and link the convergent TCR response of ARLA-specific T cells to the functional profile of these cells.

In summary, we detected disease-specific TCRs within the inflamed joints of European children and adolescents with ARLA, which will facilitate the identification of major antigen targets in this diseases. The ARLA-specific T cells displayed continuous proliferation, potentially triggered by localized TCR signaling, and demonstrated a pathogenic effector profile akin to peripheral T_PH_ cells, characterized by the expression of CXCL13 and IFN-γ. Consequently, T_PH_ cells should be acknowledged as the primary pathogenic Th cell subset in the inflamed joints of individuals with ARLA.

## Methods

### Patients

#### Sex as a biological variable.

Our study examined male and female patients, and similar findings are reported for both sexes.

#### Study cohort.

Patients were enrolled in a prospective cohort study aimed at investigating adaptive immune responses within the inflamed joints of patients affected by different forms of childhood arthritis. Enrollment occurred at the University Children’s Hospital Würzburg between January 2018 and December 2023. All children diagnosed with any form of arthritis who underwent diagnostic or therapeutic joint puncture were eligible for inclusion in this study. Patients with JIA served as a disease control. A core set of clinical and demographic data (age, sex, age at onset of arthritis, previous and current medication, ANA status, and *Borrelia* serology) was collected from all patient at the time point of study inclusion. Follow-up data of patients with ARLA was retrieved from retrospective chart review. While SF samples were obtained from all patients, matched peripheral blood samples were obtained from a subset of patients. Additionally, synovial tissue samples were obtained from 3 patients with ARLA undergoing diagnostic biopsy or synovectomy. Peripheral blood samples were additionally obtained from healthy, age-matched individuals in a control group who did not have an underlying rheumatic or infectious disease. ARLA was defined as arthritis with serological evidence of late-stage *Borrelia burgdorferi* infection (*Borrelia burgdorferi* IgG ELISA positive, IgG immunoblot with at least 5 positive bands; recomWell Borrelia IgG and IgM, and recomLine Borrelia IgG and IgM from Mikrogen Diagnostik, Neuried, Germany) that did not respond to at least 2 cycles of antibiotic treatment, including a combination of 4 weeks of amoxicillin, and/or 4 weeks of doxycycline, and/or 2 weeks of cefotaxime/ceftriaxone. SF samples from patients with ARLA were obtained after completion of antibiotic treatment. JIA was defined according to the ILAR classification criteria and only patients with JIA without serological evidence of prior *Borrelia* infection (IgG- and IgM-negative by ELISA) were included in this study as disease controls. The data for the JIA disease control group was partially published in a previous study (24). All patients diagnosed with ARLA who were referred to the Pediatric Rheumatology Clinic at the University Children’s Hospital Würzburg during the study period were included in this analysis (*n* = 11). Additionally, 2 other centers (University Children’s Hospital Leipzig, Lepzig, Germany and Children’s Hospital, Vivantes Klinikum im Friedrichshain Berlin, Berlin, Germany) contributed 1 patients with ARLA each to this study by providing biomaterials and clinical data. HLA typing was performed on DNA extracted from peripheral blood or buccal swabs by an Illumina MiSeq based next generation sequencing method at the DKMS Life Science Lab (Dresden, Germany). The distribution of selected HLA-DRB1 allele within the North American cohort of patients with ARLA was obtained from a previous study (8).

### Sample preparation

Mononuclear cells were isolated from SF or peripheral blood using Ficoll density-gradient centrifugation. Mononuclear cells from synovial tissue were acquired through mechanical disaggregation, followed by Ficoll density-gradient centrifugation. Cells were preserved in 10% Fetal Calf Serum (FCS, Gibco) 10% DMSO (Roth) and stored in liquid nitrogen until further use.

### IHC and MACSima Imaging Cycling Staining

Synovia were fixed in 4% paraformaldehyde and embedded in paraffin after dehydration in alcohol. IHC was performed using standard diagnostic protocols. For MACSima Imaging Cycling Staining (MICS), iterative staining and image acquisition was performed on the MACSima instrument (Miltenyi Biotec). Experimental details are described in the Supplemental Methods.

### Antibodies, flow cytometry, and cell sorting

Antibodies used for flow cytometry and cell sorting are listed in the Supplemental Methods. Mononuclear cells were stained in 1 × PBS 0.5% BSA (Sigma-Aldrich) with appropriate antibodies at 4°C for 30 minutes. Intracellular staining was carried out according to the manufacturer’s instruction using Intracellular Fixation & Permeabilization Buffer (eBioscience). For detection of intracellular cytokine expression, mononuclear cells were stimulated with phorbol 12-myristate 13-acetate (PMA, 50 ng/mL; Sigma-Aldrich) and ionomycin (1 μg/mL; Sigma-Aldrich) with addition of Brefeldin A (5 μg/mL, BioLegend) for 4 hours before staining. For detection of intracellular CXCL13 expression, mononuclear cells were stimulated with CD3/CD28 Dynabeads (Thermo Fisher Scientific) at a bead-to-T cell ratio of 1:1 for 6 hours with addition of Brefeldin A for the last 4 hours. Flow cytometry data was acquired on a FACSCanto II (BD Biosciences) and analyzed with FlowJo version 10 (Tree Star). Cell sorting was performed on a FACSAria III (BD Biosciences). Alternatively, CD4^+^ T cells were purified using the EasySep Human CD4^+^ T Cell Isolation Kit (Stemcell Technologies).

### TCR-β repertoire sequencing

RNA from CD4^+^CD45RO^+^PD-1^hi^HLA-DR^+^ and CD4^+^CD45RO^+^PD-1^lo^HLA-DR^–^–sorted T cells obtained from SF and synovial tissue was purified using RNeasy Micro Kit (Qiagen). Total numbers of sorted cells from the 2 T cell populations were adjusted to the lowest cell number obtained within each patient. TCR-β repertoire sequencing was performed either by amplicon rescue multiplex PCR from a commercial provider (iRepertoire) or by multiplex PCR using an in-house pipeline and sequenced by next generation sequencing on a MiSeq platform. Experimental details are described in the Supplemental Methods.

### TCR-β repertoire analysis

Resulting sequence data were analyzed using IMGT/HighV-QUEST, ARGalaxy, Alakazam, Immunarch, and GLIPH2 (Grouping of Lymphocyte Interactions by Paratope Hotspots, version 2). Details of the analytical work flows are described in the Supplemental Methods.

### 10X Genomics Chromium scRNA-Seq

SF CD4^+^ T cells from 3 patients with ARLA (ARLA01, 02, and 05) were subjected to scRNA-Seq (10x Genomics). For ARLA01 and 05, samples were multiplexed with TotalSeqC Hashtag Oligos (Biolegend; ARLA01: GTCAACTCTTTAGCG, ARLA05: TTCCGCCTCTCTTTG) prior to preparation of single-cell suspensions. After encapsulation and barcoding (10X Genomics), cells were lysed and cDNA was prepared to create a 5′ gene expression library and a VDJ gene–enriched library for TCR repertoire analysis. Libraries were sequenced using Illumina NovaSeq 6000. Raw data were processed by CellRanger (10X Genomics) with standard settings. Resulting output files were imported in R version 4.2.1 using the Read10X function of Seurat version 4.1.1 (51). Details of the further analytical work-flows are described in the Supplemental Methods.

### Adaption of published TCR-β data

Publicly available TCR-β repertoire sequencing data sets were reanalyzed for this study. The analytical workflow is described in detail in the Supplemental Methods.

### Data visualization

Various R packages (ggplot2, ggalluvial, alakazam, immunarch, ggseqlogo, Seurat, scRepertoire, and circlize) were employed for figure generation regarding TCR-β and scRNA-Seq analysis. Additionally, Prism 10.1.0 (GraphPad) was utilized to visualize quantitative data generated from other sources.

### Statistics

Statistical analysis was performed using Prism 10.1.0 (GraphPad). Data are expressed as scattered individual values and mean ± SD. 2-tailed Student’s *t* test or 1-way ANOVA with Dunnett’s or Tukey’s multiple comparisons test were used for comparison of data sets with 2 or more continuous variables, respectively. For contingency tables χ^2^ with Yates’ correction was used. A *P* value < 0.05 was considered as statistically significant.

### Study approval

Written informed consent was obtained from the legal guardians of each participant. The study was approved by the Research Ethics Committee of the University of Würzburg (299/17) and conducted in strict accordance with the principles of the Declaration of Helsinki.

### Data availability

The values for all data points in graphs are reported in the Supporting Data Value file. Bulk TCR sequencing data is deposited in the NCBI Sequence Read Archive under BioProject ID PRJNA1054606. The single cell sequencing data discussed in this publication have been deposited in NCBI’s Gene Expression Omnibus and are accessible through GEO Series accession number GSE247675.

## Author contributions

JD and HM designed the study. JD, JF, JK, VB and IC performed experiments. JD, JF, JK, AHW, CH, CK and HJG collected data. JD, JF, JK, AHW, CH, CK, VB, HJG, IC and FE analyzed data. JD and HM drafted the manuscript, and all authors revised and finally approved the manuscript.

## Supplementary Material

Supplemental data

ICMJE disclosure forms

Supplemental tables 4-6

Supporting data values

## Figures and Tables

**Table 1:**
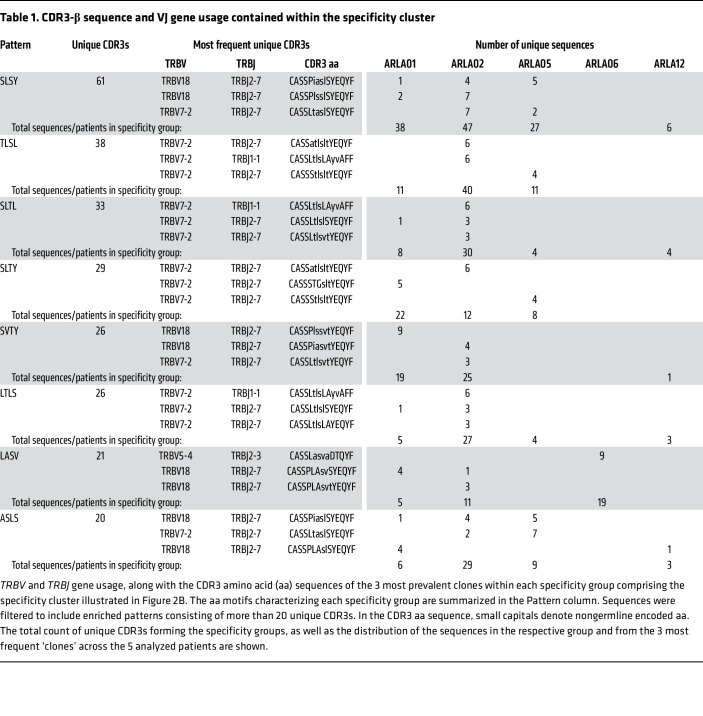
CDR3-β sequence and VJ gene usage contained within the specificity cluster

## Version 1 07/04/2024

In-Press Preview

## Version 2 09/03/2024

Electronic publication
